# Consequences of the legislation issued for nursing education in times of COVID-19 (2020-2022)

**DOI:** 10.1590/0034-7167-2023-0375

**Published:** 2024-10-07

**Authors:** Ailla Gabrielli Costa Silva, Lenira Maria Wanderley Santos de Almeida, Regina Maria dos Santos, Danielly Santos dos Anjos Cardoso

**Affiliations:** I Universidade Federal de Alagoas. Maceió, Alagoas, Brazil

**Keywords:** Education, Nursing, COVID-19, Higher Education Policy, Legislation, Education, Graduate, Educación en Enfermería, COVID-19, Política de Educación Superior, Legislación, Educación Superior

## Abstract

**Objectives:**

to analyze the legislation issued during the COVID-19 pandemic for nursing education and immediate consequences.

**Methods:**

documentary research, whose source was the legislation issued for nursing education during the COVID-19 pandemic. Thirty-two documents were analyzed and submitted to a collection instrument.

**Results:**

contradictions and consequences for nursing education following the guidelines and law of guidelines and bases were revealed. The consequences of the law affected the quality of teaching, equal access, exemption from minimum school days and course abbreviation. Regarding the guidelines, there was a lack of conditions for developing skills, in addition to the attempt to update them. Concerning internships, acting during the pandemic put students at risk, and their abbreviation prevented them from consolidating knowledge and skills.

**Final Considerations:**

remote teaching did not guarantee the quality and equality of teaching, weakened the development of skills, not taking into account nursing’s educational needs.

## RESUMEN

**Objetivos:**

analizar la legislación emitida durante la pandemia de COVID-19 para educación en enfermería y sus consecuencias inmediatas.

**Métodos:**

investigación documental, cuya fuente fue la legislación emitida para educación de enfermería en la pandemia. Se analizaron 32 documentos y se sometieron a un instrumento de recolección.

**Resultados:**

hay contradicciones/consecuencias para formación de enfermería siguiendo las directrices y la ley de directrices y bases. La ley afectó la calidad de enseñanza, igualdad de acceso, exención de jornada escolar mínima y abreviatura de cursos. En cuanto a los lineamientos, faltaron condiciones para el desarrollo de habilidades, además del intento de actualizarlas. En cuanto a las pasantías, actuar durante la pandemia puso en riesgo a los estudiantes y su abreviación les impidió consolidar conocimientos/habilidades.

**Consideraciones Finales:**

enseñanza remota no garantizaba la calidad/igualdad de la enseñanza, debilitó el desarrollo de habilidades, al no tener en cuenta las necesidades educativas de enfermería.

## INTRODUCTION

With the declaration of the Coronavirus Disease 2019 (COVID-19) pandemic by the World Health Organization in 2020, it was necessary to adopt measures such as physical distancing, social isolation and quarantine to prevent and control the virus transmission. Social distancing required non-in-person activities for services considered non-essential, such as in-person classes in educational institutions, practical activities and internships. In response to the social distancing measures adopted and the need to continue activities, the Ministry of Education (MEC – *Ministério da Educação*) regulated the use of digital technologies and tools to carry out classes^(1)^.

Before the pandemic, Digital Information and Communication Technologies (DICT) were already used, especially in distance learning (DL) courses. This modality has been regulated since 2017 and is characterized by technological mediation between professors and students, with emphasis on activities taking place in different places and times^(1)^. During the pandemic, what happened was the application of emergency remote teaching, which differs from DL, as it is the transposition of in-person classes to the virtual environment, even requiring regulation by the government^(2)^.

Higher education is governed in Brazil by the Law of Guidelines and Bases of National Education (LDB - *Lei de Diretrizes e Bases da Educação Nacional*), enacted in 1996, which sets out the bases of education in Brazil, such as higher education. Higher Education Institutions (HEIs) develop and execute the pedagogical proposal based on national guidelines^(3)^. The Brazilian National Curricular Guidelines for the Undergraduate Nursing Course (DCNEnf - *Diretrizes Curriculares Nacionais do Curso de Graduação em Enfermagem*) were published in 2001 and provide general and specific skills to be developed by students during graduation. They recommend comprehensive training, based on active methodologies and critical reflective professionals focused on the Brazilian Health System (SUS - *Sistema Único de* Saúde)^(4)^.

The application of remote teaching for nursing required that the replacement of classes by virtual means be standardized, considering that using DICT as a replacement for in-person teaching was not foreseen in LDB or DCNEnf^(1)^. Thus, the relevance of this study translates into the possibility of producing knowledge about the circumstances of training health workers amidst the pandemic, with systematized recording of possible consequences for undergraduate nursing education.

Therefore, the study was guided by the following guiding question: what regulations were established for undergraduate nursing education resulting from governmental and institutional measures adopted during the COVID-19 pandemic and their immediate consequences for preserving the quality of professional training based on LDB and DCNEnf?

## OBJECTIVES

To analyze the legislation issued during the COVID-19 pandemic for nursing education and immediate consequences.

## METHODS

### Ethical aspects

As this is a documentary study whose sources are in the public domain and freely accessible, there was no assessment by a Research Ethics Committee, although ethical principles were respected throughout the work path.

### Study design

This is descriptive and documentary research with a qualitative approach, being a type of study in which information comes from documents, with the aim of understanding a phenomenon, and which is carried out in two phases: preliminary analysis of context and actual analysis of its content^(5)^. The research documentary *corpus* consisted of 32 normative documents on teaching during the pandemic. Standard for Reporting Qualitative Research (SRQR) recommendations, an instrument from Enhancing the QUAlity and Transparency Of health Research (Equator), were followed.

### Methodological procedures

Sources of information were identified on official websites of bodies and institutions, such as the Federal Government, Ministry of Health, MEC, Brazilian National Health Council (CNS - *Conselho Nacional de Saúde*), Brazilian National Education Council (CNE - *Conselho Nacional de Educação*), Brazilian Nursing Association (ABEn - *Associação Brasileira de Enfermagem)* and Federal Nursing Council (COFEN - *Conselho Federal de Enfermagem*). The identified bodies and institutions were included because they issue legal standards, opinions and recommendations for teaching in general and nursing. The search was carried out from October 2022 to April 2023. The documents were searched by the keywords “Distance/Remote/Emergency Education”; “Higher Education/Teaching”; “Nursing Teaching”.

Initially, 68 regulations were found, including laws, resolutions, ordinances, recommendations and opinions. Then, the documents found were subjected to inclusion criteria, such as meeting the research question and objectives, in addition to the time frame of the pandemic. To be included, documents needed to be dated, signed by an authority and establish standards or recommendations to be obeyed or observed. Furthermore, extension regulations, as they did not add new elements to teaching during the pandemic, and laws that did not deal with higher education and/or nursing education were excluded. Time frame was based on the declaration of COVID-19 as a public health emergency of international concern and the closure of the emergency between February 3, 2020 and April 22, 2022^(6)^.

Selected documents were submitted to a data collection instrument built based on the points covered in LDB and DCNEnf. The instrument collected information on type of document, body, publication date, document description, keywords, correlation and obedience to LDB (law articles and excerpts from documents), correlation and respect for DCNEnf (guideline topics and excerpts from documents). The information extracted through the instrument was organized in a Microsoft Excel® spreadsheet.

The documents were cataloged and systematized, depending on whether the content proposed changes to LDB or DCNEnf that would have an impact on quality of professional training with immediate consequences for training in time frame. The result of this analysis was compared with the literature, creating a dialogue with regulations and generating three categories of analysis.

## RESULTS

**Figure 1:**
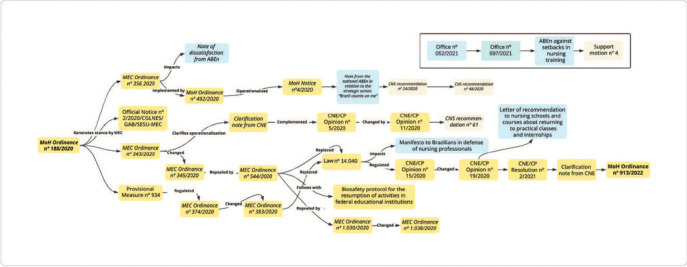
Existing relationships between regulations

Based on the analysis of the 32 selected public documents, the following categories emerged: Regulations relating to the Law of Guidelines and Bases of National Education and its consequences (Subcategories General aspects of teaching and Higher education); Regulations relating to the Brazilian National Curricular Guidelines for the Undergraduate Nursing Course and their consequences; and Regulations for supervised internship and its consequences. After analyzing the documents, it was possible to infer existing relationships between them, presented in the form of a conceptual map in Figure 1.

### Regulations relating to the Law of Guidelines and Bases of National Education and its consequences

#### Subcategory 1: General aspects of teaching

The analysis of regulations published for nursing education begins with the discussion on the 1996 LDB, already in its Article (Art.) 1, in which many of regulations only brought the link between education and the world of work. MEC Ordinance 356/2020/,by prioritizing the insertion of students in the fight against the pandemic, highlighted only the market need, ignoring that students were in training and that, in addition to being at risk, they would not provide safe and adequate care to the population, as reinforced by ABEn, when positioning itself contrary to MEC^(3, 7, 8)^.

Art. 1 Students regularly enrolled [...] in the final year of nursing courses are authorized [...] the possibility of carrying out the mandatory curricular internship in basic health units, emergency care units, hospital networks and communities to be specified by the Ministry ofHealth while the public health emergency situation resulting from COVID-19 (coronavirus) lasts^(7)^. (free translation)

The principles of national education, especially the guarantee of quality standards, were quite recurrent, either by defending their maintenance or by the possibility of affecting the quality of education recommended by LDB. This concern was mentioned especially in opinions published by CNE in defense of use of digital methodologies. Replacing in-person classes with digital classes did not provide conditions that ensured the fulfillment of these purposes by replacing classes, as was recommended^(3, 9, 10, 11)^:

It is important to highlight CNE’s statement in its Note that, in the process of reorganizing school calendars, it must be ensured that the replacement of classes and the carrying out of school activities can be carried out in a way that preserves the quality standard provided for in item IX of article 3 of LDB and in item VII of article 206 of the Federal Constitution^(10)^. (free translation)

Another point addressed in the principles was about equal conditions of access and permanence at school. With large-scale application of remote teaching, a debate began about students’ access to quality equipment and internet. This is evidenced in CNS recommendation, which states that the application, even if theoretical, of the DL modality did not include vulnerable students^(3, 12)^.

Even in the case of theoretical activities, it is necessary to guarantee the different needs of students. The imprecision in that legal standard regarding which resources, specifically, the scope ofthe same refers to and on the ways of monitoring activities in the DL modality, which require electronic equipment, makes it impossible to include students who reside in areas of greater vulnerability and who do not have financial resources^(12)^. (free translation)

Art. 9 was observed in the federal government’s regulations, as the Union must organize, maintain and develop HEIs, in addition to collecting, analyzing and disseminating information about education, which was addressed in Circular Letter 2/2020/CGLNES/GAB/SESU/SESU-MEC. Finally, the union’s role was fulfilled by issuing general rules on undergraduate courses^(3, 13)^. Art. 9 also mentions CNE as a body with regulatory and supervisory functions and permanent activity. A point that was fulfilled and could be observed in the opinions issued and in Law 14,040, which delegated implementation guidelines to CNE, which was later implemented with CNE/CP Opinion 11/2020^(3, 10, 11, 14)^.

Art. 12 was addressed by federal regulations to ensure that HEIs could develop and execute their pedagogical proposal. Students’ rebound could be seen in CNE Opinion 5/2020, stating that HEIs should consider “building a rebound program, if necessary so that all children can develop”^(3, 10)^. Professors also complied with Art. 13 in what they were responsible for, with CNE/CP Opinions 5/2020 and 11/2020 being some of the documents that contained the points of the aforementioned article and the teaching role^(3, 10, 11)^.

#### Subcategory 2: Higher education

Entering the specific chapter for higher education, in Art. 43, the higher education purposes were described. One of them, the training of graduates, could be understood as justification for Provisional Measure (PM) 934/2020, which allowed courses to be shortened. Encouraging knowledge about problems in the present world was observed with the promotion of prevention campaign in Circular Letter 2/2020/CGLNES/GAB/SESU/SESU-MEC^(3, 13, 15)^.

Non-in-person activities to reorganize the calendar were based on Art. 47 MEC Ordinance 343/2020 used the need to comply with school days as a justification to allow the replacement of classes in digital media: “Suspended academic activities must be fully restored for the purpose of complying with the school days and class hours established in the legislation in force”^(3, 9)^.

However, PM 934/2020 later violated MEC’s prescription, by exempting the fulfillment of 200 minimum school days, and it was only necessary to comply with the minimum workload (WL), as established by Law 14.040/2020. PM 934/2020 also changed the requirements for abbreviating courses, establishing that it would be enough to fulfill 75% of the WL allocated to the internship, as can be seen below^(3, 14, 15)^:

Higher education institutions are exceptionally exempt from the obligation to observe the minimum number of days of effective academic work [...]. In the hypothesis referred to in the caput, the higher education institution may shorten the duration of [...] courses, as long as the student, observing the rules to be edited by the respective education system, complies, at least [...] II - seventy-five percent of the workload of the mandatory curricular internship of nursing courses^(15)^. (free translation)

Universities’ autonomy in Art. 53 was guaranteed in most of the attributions, with the exception of adherence to action “Brazil counts on me”, which occurred obligatory, with HEIs having to communicate to students, with CNS suggesting optional participation between HEIs and federative entities. The assignments were made under democratic management, as required by Art. 56^(3, 16, 17)^.

According to Art. 55, the Union is responsible for ensuring sufficient resources for the maintenance and development of HEIs, with expenses for maintenance and development of teaching being defined in Art. 70. Regarding providing equipment necessary for teaching, CNS, in its recommendation 24/2020, proposed that HEIs guarantee Personal Protective Equipment (PPE) to students participating in action “Brazil counts on me”^(3, 17)^.

The Federal Government’s stance of immediately opting for remote teaching at the beginning of the pandemic is related to Art. 80 of LDB, which states that public authorities “will encourage the development and delivery of distance learning programs, in all levels and modalities of teaching, and continuing education”. This fact explains MEC’s first decision to replace in-person classes with digital classes in MEC Ordinance 343/2020^(3, 9)^.

### Regulations relating to the Brazilian National Curricular Guidelines for the Undergraduate Nursing Course and their consequences

DCNEnf are those who guide nursing training in the country, bringing skills to be developed, which occurs as students have contact with practice scenarios. And what was seen in the regulations was the large-scale application of remote teaching, in contradiction to what entities defended about nursing training. Therefore, the MEC and CNE documents that instituted the application of non-in-person methodologies for classes, practices and curricular internships ended up interfering with the development of necessary skills for students, as stated by ABEn and CNS^(4, 9, 10, 18)^.

We demand that the Ministry of Education (MEC) comply with the Brazilian National Curricular Guidelines (DCNs) for training nursing professionals in in-person, because it is impossible to teach care without touch, without embracing pain and fears in practice TOGETHER with users so that critical sense, thorough observation, carrying out procedures and an ethical and problem-solving attitude are developed^(18)^. (free translation)

However, even in conditions of a pandemic or other health emergency, virtual resources do not completely supply health work and, therefore, do not cover the conditions in which most professional skills and competencies must be developed in internship activities in real situations^(12)^. (free translation)

The next point is the curricular contents, which explain the contents that must be covered during graduation to promote the development of skills. Law 14,040, by exempting the minimum number of school days, conditioned the non-compromise of content essential to the profession, acting in a way to promote the recommended teaching when analyzing only^(4, 14)^:

Higher education institutions are exempted, on an exceptional basis, from the obligation to observe the minimum number of days of effective academic work [...], as long as [...] II - there is no harm to the essential contents for the exercise of the profession^(14)^. (free translation)

In 2021, CNE presented a new DCNEnf proposal to ABEn and CNS. According to letters published by ABEn and COFEN, in addition to the ABEn manifesto and the CNS motion, the proposal distorted the nursing degree. The documents pointed out conceptual errors, empty concepts, the possibility of a hybrid modality, no definition of curricular internship and practical activities, in addition to the lack of a clear theoretical framework. It was observed in the documents the defense of quality nursing education that would allow the development of skills, with reference to the SUS, the educational and social role of nurses^(4, 19, 20, 21, 22)^.

Research and extension are part of the list of complementary activities recognized by DCNEnf, and the virtual carrying out of research and the regulation of extension activities are recommended, with a suggestion to associate research and extension with internship replacement^(11)^.

The course organization is ensured in DCNEnf with the construction of a pedagogical project, with comprehensive training, articulation between teaching, research, extension and care, and methodologies that articulate learning to learn and learning to do. CNE, in Opinion 19/2020, recognized that replacing by digital classes represented a limitation in using methodologies and technologies aimed at the virtual environment. The possibility of suppressing professional practices ended up interfering with the interaction of tutors and students with the population, as practice favors theory-practice integration during the course. As for the topic of monitoring and assessment, CNE/CP Opinion 5/2020 brought the need to assess teaching in the period^(4, 10, 23)^.

Finally, the Course Completion Work (CCW) is standardized in DCNEnf, in the course organization, as a requirement for completing the course, preparing a work under teaching guidance. CNE advised that activities related to CCW should occur in person, requiring regulation^(4, 10)^.

### Regulations for supervised internship and its consequences

The last category identified corresponds to supervised internship, which crosses both regulations. The internship standards will be established by education systems in accordance with Art. 82 of LDB, in compliance with federal law. In DCNEnf, there is an obligation to include an internship in healthcare services with at least 500 hours in the last two semesters. Initially, the internship was prohibited from being replaced by digital means, with MEC Ordinance 343/2020, before being carried out as an action to combat the pandemic, with MEC Ordinance 356/2020^(3, 4, 7, 9)^.

ABEn promptly expressed its opposition to the use of students as a workforce, as did CNS. The federal government, through PM 934/2020, allowed the shortening of the course with the completion of 75% of the mandatory internship WL. CNE, through CNE/CP Opinion 5/2020, positioned itself in favor of the adoption of non-in-person activities for internships, which was accepted by MEC in MEC Ordinance 544/2020^(8, 10, 15, 17, 24)^.

## DISCUSSION

The first point to analyze is the diversity of nomenclatures used, such as DL, digital classes, remote activities, DICT, remote learning and teaching. The terms “DL” and “remote teaching”, which are sometimes used as synonyms, refer to different modalities. DL is characterized by systematization of teaching-learning, methodology, strategies and specific planned materials; model aimed at students, professors and tutors; monitoring by professors or tutors; synchronous and asynchronous activities; use of resources on different platforms. Emergency remote teaching is characterized by the physical distancing of students and professors, temporary nature and mainly by the transition from in-person to virtual teaching with the aim of mitigating impacts and losses on student learning^(2, 25)^.

LDB and DCNEnf regulate and bring the principles to the quality standard of education nationally. Fernandes *et al.* ^(26)^, when analyzing the federal normative acts adopted during the pandemic, reflected that sometimes the execution of essential tasks that resulted in quality was limited or not included. They pointed out contradictions in the published ordinances, which exempted the minimum number of school days as long as WL was completed and, at the same time, allowed the abbreviation of courses. Another contradiction was the application of remote teaching to protect students and professionals while calling them to work during the pandemic. The authors argued that remote teaching did not bring conditions to fulfill the teaching purposes established by LDB, and they also contradict DCNEnf when they allowed practical activities at a distance.

A study by Fernandes *et al.* ^(26)^ assessed that the measures aimed to supply professionals for healthcare services without worrying about the quality of training. Teaching and practice, combined with the world of work, provide students with the opportunity to form critical thinking about their performance and develop skills. Taking training away from reality, experienced in practices, leads to only theoretical training, without problematizing social issues, without adopting the SUS as a coordinator. By leaving it up to HEIs to make tools available, the budget of public HEIs was not considered, and the Union was not held responsible for guaranteeing resources. The authors concluded that remote teaching did not meet the needs of nursing training, resulting in the maintenance of quality proposed by LDB, in addition to addressing other aspects of the law.

Equal access conditions provided for in LDB saw a situation never imagined in the exceptional nature of the pandemic. Suspension of classes for long periods and an abrupt change to the virtual environment collided with socioeconomic inequality in the country, as public-school students especially have difficulty accessing the internet and the necessary technologies. The impacts increased existing inequalities, in addition to uneven learning levels. The urgency justified in the measures taken should not be used to transform the right of everyone to education into the right of the few who have resources^(27)^.

Access to education was not just about having access to the internet; it was also necessary to have equipment and digital literacy to use the tools adopted. Access democratization should be linked to a policy of expanding digital access to the population. This would allow the appropriate use of technologies, a fact that did not occur and is not currently discussed. These aforementioned changes affect everyone, and, for professors, there is also the requirement and overload of WL, generating occupational stress and affecting physical and mental health^(28)^.

CNE, as a body with normative, supervisory and permanent activity in educational activities, gained prominence with the opinions regulating teaching during the pandemic. Mascarenhas and Franco^(29)^ analyzed Opinion 5/2020, which was the first to suggest non-in-person activities. The opinion referred to education as the fulfillment of hours and content, which applied to MEC Ordinance 343/2020, disregarding the social role of the school and exposing a technical nature adopted by the government, ignoring the real situation of education in the country. Finally, the authors considered the opinion fragile, discriminatory and perverse towards public school students.

Extension, during the pandemic, suffered from social distancing measures. Some were initially suspended as they were unviable due to social isolation. During the implementation of emergency remote teaching, there was an acceleration in teaching and research production with adaptation to actions to combat the pandemic. Combat actions involved testing for COVID-19, educational materials, participation in the vaccination campaign, among others^(28, 30)^.

One of the points that required the most regulation was the mandatory internship. Curricular internships in health require an in-person presence that was not possible due to the conditions imposed by the pandemic. The measures adopted allowed students undergoing training during the pandemic without guaranteeing safety or the training process. Subsequently, replacement was allowed in digital media that appeared in DCNEnf, approved by the collegiate. Legal acts demonstrated concern in addressing service deficiencies, without considering other existing structural difficulties, such as lack of PPE. Issues such as patient safety, care with fewer errors and alignment with HEIs were also left aside^(31)^.

Practice and internship scenarios need to be preserved as essential teaching spaces in learning and theory/practice articulation, seen in DCNEnf. The diversity of scenarios also contributes to building professional identity, producing care and articulating knowledge with the population’s health reality. The abbreviation of courses, upon completion of 75% of the internship WL, also came to speed up training to meet the service demands. At the same time, it prevented the consolidation of theoretical knowledge, development of technical skills and attitudes towards students’ professional future^(31)^.

Analyzing action “Brazil counts on me” and the course abbreviation, Mata *et al.* ^(32)^ highlighted that training guidelines state that professionals must meet social health needs in a comprehensive and humanized manner. Training that achieved this success among graduates was already considered difficult before the pandemic, due to the inconsistency of discourse with practice, a fact observed by students, an inconsistency that tended to deepen with the anticipation of the conclusion^(32)^. Teaching must be strengthened, not weakened, in line with the SUS theoretical assumptions, thus implementing generalist training instead of focusing on assistance and hyperspecializations.

Even before the pandemic, the need for supervised internships was already reinforced as a developer of general skills. Benito *et al.* ^(33)^ described the general competencies of DCNEnf and articulated them with supervised internship. Complete development is not completed during training, being a professional commitment, however, the authors pointed out the internship and practices as strengthening, promoting and expanding skills, with skill-based learning being a pedagogical process of greater transformation that receives information passively. There is also the role of students as a change-maker who will act by shaping the reality to be faced by the healthcare team.

Other points to be analyzed did not have a direct impact on regulations, but are issues inherent to DL and currently also to remote teaching for nursing. Therefore, it is also worth discussing, since, even though it is not the modality used in the pandemic, using DICT in nursing is not new and has been discussed for some time by theorists in the field.

The year 2020 was considered the international year of nursing, with the launch of campaigns such as Nursing Now, which took on another dimension with the pandemic. One of the points of the campaign was the valorization of the profession, in which nurses are understood as leaders, with a role of excellence, providing care ethically and in a qualified manner. Therefore, one of the ways to value and promote this profile is through strengthening education and professional development. However, what was seen were complaints of terrible working conditions for nursing, illness of professionals and the massive adoption of remote teaching in training^(34)^.

Professional autonomy is related to the incorporation of nursing as a science and the Nursing Process. Historically, autonomy was linked to training, with Florence bringing authority to nurses within their field of knowledge, until the moment when training was modified to meet financial and medical interests. When it was possible to mischaracterize nursing as a science, there was a loss of autonomy for nurses, who were reduced to reproducing orders from the medical profession. Nurses need to take control of their science, which is directly related to training that promotes autonomy and the application of the Nursing Process. Furthermore, there is a need for legislation focused on nursing and managers who defend professional performance so that nurses’ praxis is recognized and valued^(35)^.

A problem generated by offering courses in distance learning is the advancement of privatization of education, raising doubts about social commitment, especially the training ordered for the SUS, as guided by the Federal Constitution of 1988, the Organic Health Law and DCNEnf^(36)^.

The alternative found for developing practical activities in laboratories was to replace theoretical classes, not carry them out, or the class to take place with the professor in the laboratory and the student at home synchronously. The authors also brought out the decrease in the number of students per practice group, which leads to uncertainty in the quality of the activities offered, both in terms of the impact of non-compliance and the replacement of practice by technological mediation^(37)^.

Even with Federal Law 14,040/2020 conditioning the exemption from minimum school hours to maintaining the expected WL and without compromising content, what happened in reality was different. Baixinho and Ferreira^(38)^ identified in students’ speech that reducing hours was a difficulty encountered during the internship, generating concern in relation to learning capacity and clinical decision-making, in addition to professional development.

Nursing care requires the presence and use of the senses, being a central concept in several nursing theories. Today, there is a debate about presence in a digital world that also extends to nursing. Being with patients, which encompasses touching, listening and understanding, became a challenge to the essence of care during the pandemic. If, for nurses, presence is essential for carrying out nursing care, why would it be different for students? Just like professionals, students, in addition to developing skills, need to be prepared to work with people in any circumstance in life and even more so in conditions of suffering so that merely cognitive teaching does not achieve this objective, nor does it provide safe practice, supportive, competent and ethical. This leads to reflection on the need for in-person teaching and exercising presence as care^(25, 39)^.

### Study limitations

Limitations included having been carried out from the perspective of documentary research, restricted to federal regulations, to some bodies and institutions, limiting the analysis to legal issues, without investigating the implementation of changes, perceptions of the teaching offered and possible repercussions on professors’ and students’ health.

### Contributions to nursing

The contributions of this study are provision of relevant systematized material, in addition to analysis of the repercussions of the applicability of normative instruments for nursing education during the pandemic based on LDB and DCNEnf.

## FINAL CONSIDERATIONS

Nursing education researchers point to the wide application of remote teaching as a factor that promotes weaknesses in the development of skills and the consolidation of theoretical knowledge. Some of the relevant aspects that contributed to this condition were: the replacement of mandatory practice and internship scenarios with digital technologies; the different forms of access to remote teaching that students and professors were subjected to, both due to the need to acquire quality equipment and internet and the appropriate structure, skills and knowledge to use platforms and resources; and the discussion about the training quality standard proposed by LDB and its actual implementation amidst the pandemic.

In addition to the measures taken as a result of the pandemic, there was an attempt by CNE to update DCNEnf for nursing, situation that mobilized nursing bodies to point out theoretical and methodological weaknesses, in addition to the inability to implement hybrid teaching due to the nature of the profession and the absence of SUS as the organizer of training in the proposal presented.

It is worth rethinking the use of DICT as a replacement for in-person teaching, due to the possibility of a new pandemic occurring. Managers and professors must work on plans to incorporate technologies as a pedagogical aid, not as a replacement, considering that it is learning that requires contact, bonding, touch and, therefore, develops learning about intersubjective care relationships.

On the other hand, it is important to establish biosafety committees to build coordinated action based on protocols that ensure adequate protection for in-person teaching and to articulate actions in which the university, through the nursing course, can collaborate safely with the population, asserting its ethical and social commitment. Thus, it will also be cooperating with the familiarity of professors and students with technological teaching tools, without limiting the development of skills and strengthening the construction of new alternatives.

## Supplementary Material

0034-7167-reben-77-05-e20230375-suppl01

## Data Availability

https://doi.org/10.48331/scielodata.TSZQM9
