# Supplementary material for: Consequences of the legislation issued for nursing education in times of COVID-19 (2020-2022)
Source: Rev Bras Enferm. 2024 Oct 7;77(5):e20230375. doi: 10.1590/0034-7167-2023-0375 (PMC11458146; doi:10.1590/0034-7167-2023-0375)
Supplement: 0034-7167-reben-77-05-e20230375-suppl01 [file 0034-7167-reben-77-05-e20230375-suppl01.pdf]

normativa,tipo_de_documento,orgao_publicacao,data_publicacao,descricao_do_documento,palavras-chave,ldb_
Oficio Circu	Limpeza d	Evitar aglo	Atencao a	Competen	Promover atividades educativas sobre higiene e etiqueta
Portaria M	Atividades	Estagio e p	Competen	Estagio Cu	Organizaca	Dever da U	""As ativic	""Autoriza	""Â§ 3o Fic
Nota de Es	EAD	Atividade	Competen	Organizaca	""2. no exe	""as instituicoes de educacao superior poder
Portaria M	Atividades	Estagio e p	Competen	Estagio Cu	Organizaca	""Â§ 3o Fica vedada a aplicacao da substituic
Portaria M	Brasil cont	enfrentam	Competen	Estagio Cu	Organizaca	""Â§ 5o A	""Â§ 6o A realizacao do estagio ol
nas Diretrizes Curriculares Nacionais do Curso e nao relacionadas ao COVID-19 (coronavirus), que deverao ser curs
Portaria M	Brasil cont	enfrentam	Competen	Estagio Cu	Organizaca	""Art. 15. /	""Art. 17. Para a execucao do disp
Nota de de	Brasil cont	Atendimer	Formacao	Competen	Estagio Cu	Organizaca	""Entretanto, sabemos que os est
Edital MS n	Brasil cont	enfrentam	Competen	Estagio Cu	Organizaca	""2.1.2. A	""5.2.3.3.1. A carga horaria cumpı
Medida Pr	Carga hora	Abreviacac	Competen	Estagio Cu	Organizaca	""Paragrafo unico. Na hipotese de que trata
Portaria M	Competen	Estagio Cu	Organizaca	""Art. 1o Ficam autorizadas as instituicoes de ensino pertencentes a
Nota da AE	Brasil cont	Formacao	Abreviacac	Competen	Estagio Cu	Organizaca	""entende	""Destaca-	""Quanto a
Portaria M	Competen	Estagio Cu	Organizacao do Curso,"""Art. 1o Ficam autorizadas as instituicoes de ensino per
Recomend	recomend	EPI	apoio	hierarquiz	Competen	Estagio Cu	Organizaca	""15) Reco	""5) A qua
Parecer CN	EAD	Reorganiz	Estagio,"A	Competen	Estagio Cu	Organizaca	Avaliacao,	""Tendo c	""como m
atividades	""Neste sentido, as avaliacoes e exames de conclusao do ano letivo de 2020 das escolas deverao levar
Portaria M	Atividades	Estagio e p	Competen	Estagio Cu	Organizaca	""O MINIS	""Â§ 1o As	""Â§ 2o As	""Â§ 3o No
Recomend	Brasil cont	enfrentam	EAD	abreviacac	Competen	Estagio Cu	Organizaca	""As norm	""Para isso
Protocolo	(p. 14)	""Isso post	""Recome	"" entre ou	""Atencao especial deve ser voltada a equipe responsave
Parecer CN	EAD	Reorganiz	Estagio,"A	Competen	Estagio Cu	Organizaca	Avaliacao,	""Diante d	""Alem dis
Lei no 14.0	e altera a l	abreviacac	EAD	Repasse fi	Carga hora	Competen	Conteudos	Estagio Cu	Organizaca
Manifesto	Ensino pre	Competen	Organizacao do Curso,"""Ao Ministerio da Educacao (MEC), cobramos a observa
a observacao minuciosa, a realizacao dos procedimentos e a atitude etica e resolutiva, que salvam tantas vidas e p
Recomend	investimer	acao articu	""Ao Gove	""e) A definicao de me	""c) Os inv	d) Os inves	"	EAD
observadas	""como ga	""Tendo c	""como m	""Neste se	""Por isso,	""A gestao	""Ja ha um	""A substit	""No ambi
Portaria M	atividade r	Competen	Estagio Cu	Organizaca	""O MINIS	""Â§ 1o Se	II - a dispo	e III - a rea	""Art. 2o C
Portaria M	atividade r	Competen	Estagio Cu	Organizaca	""O MINIS	""Art. 1o A	""Art. 2o C	""Art. 3o	ou II - conc
Parecer CN	EAD	Reorganiz	Estagio,"A	Competen	Estagio Cu	Organizaca	Avaliacao,	""Tendo c	""Â§ 2o De
Carta de re	EPI	Vacinacao	Competen	Estagio Cu	Organizaca	""Ressalta-se que o retorno de atividades pra
Oficio no 0	EAD	fragilidade	retrocesso	Competen	Conteudos	Estagio Cu	Organizaca	""A minuta	""e necess
Oficio no 6	EAD	fragilidade	retrocesso	Competen	Conteudos	Estagio Cu	Organizacao do Curso,"""Assim, e
cientificas	""elaborac	""Definicao clara do estagio curricular supervisionado (ECS) que deve ser oferecido ao final
ABEn contr	EAD	fragilidade	retrocesso	Competen	Conteudos	Estagio Cu	Organizaca	""Assim a	""Em confı
Mocao de	EAD	fragilidade	retrocesso	Competen	Conteudos	Estagio Cu	Organizaca	descaracte	""
Resolucao	Competen	Estagio Cu	Organizaca	Avaliacao,	o disposto [..]no Â§ 1	""Art. 1o C	""Â§ 1o To	""II - adota
Nota de es	""4. Dessa forma, o Conselho Nacional de Educacao, em consonancia com o disposto na Resolucao CN

artigos,dcnenf_topicos,trecho_de_justificativa
ı respiratoria
:a vedada a aplicacao da substituicao de que trata o caput aos cursos de Medicina bem como as praticas profission
n considerar a utilizacao da modalidade EaD como alternativa a organizacao pedagogica e curricular de seus curso:
:ao de que trata o caput as praticas profissionais de estagios e de laboratorio."""
brigatorio [...] nao desobriga o aluno de cumprir a carga horaria prevista para o estagio em outras areas, caso menc
adas normalmente pelo aluno de acordo com o projeto pedagogico do curso ao qual o aluno esta matriculado e na
osto nesta Secao, cabera as IES com cursos de graduacao em Medicina, Enfermagem, Fisioterapia e Farmacia:"""
udantes, sejam de Enfermagem ou das demais profissoes citadas, mesmo no ultimo ano do curso, estao em proce:
rida pelos alunos previstos no item 5.2.3.3., no ambito da Acao Estrategica de que trata este Edital, podera ser utili
caput , a instituicao de educacao superior podera abreviar a duracao dos cursos de Medicina, Farmacia, Enfermag
o sistema federal de ensino, definidas no art. 2o do Decreto no 9.235, de 15 de dezembro de 2017, em carater exc
s Instituicoes de Ensino Superior (IES), e imprescindivel que o seguro de saude em favor do aluno"""
rtencentes ao sistema federal de ensino, definidas no art. 2o do Decreto no 9.235, de 15 de dezembro de 2017, em
""4) Garantam ao grupo de estudantes que venham a aderir a acao estrategica â€œO Brasil Conta Comigoâ€, todo
""Neste se ""Por isso, ""A gestao ""Ja ha um ""A substit ""No ambito da oferta da educacao superior nao presen
em conta os conteudos curriculares efetivamente oferecidos aos estudantes,"" (p. 20)"
que se refere as praticas profissionais de estagios ou as praticas que exijam laboratorios especializados, a aplicaca
""Entretanto, de maneira tambem contraria a Lei de Estagio, no dia 01 de junho de 2020, foi homologado o Parec
l pela limpeza, alem da capacitacao e do fornecimento de EPIs, insumos e materiais de limpeza contribuem para s
""area de (""No ambito da oferta da educacao superior nao presencial, deverao ser adotadas e normatizadas, pa
""Paragraf ""Art. 3o A e II - nao h ""Paragraf ""Â§ 1o Pc ""Â§ 2o Na hipotese de que trata o caput deste artigo, a
ancia das Diretrizes Curriculares Nacionais (DCNs) para formacao de profissionais de enfermagem com a modalidac
reservam a saude de milhoes de pessoas"""
Reorganiz: Estagio,"A Competer Estagio Cu Organizaci Avaliacao,"""Paragrafo unico. As Instituicoes de Educaca
to da oferta da educacao superior nao presencial, deverao ser adotadas e normatizadas, para essa modalidade, ati
""Â§ 2o No que se refere as praticas profissionais de estagios ou as praticas que exijam laboratorios especializado
dicoes sanitarias locais que tragam riscos a seguranca das atividades letivas presenciais."""
""Â§ 2o A ""Art. 1o A ""Paragraf "" â€" ado (p. 15) ""V â€" ad ""II â€" adotar a substituicao de atividades pı
ticas e estagio, em cenarios reais de assistencia, deve ter foco em processos formativos que garantam a qualidade
""Ainda, sobre o estagio curricular remete a seu desenvolvimento ao longo do curso desconsiderando que se trat
de interesse do COFEN que a formacao do enfermeiro seja pautada em pressupostos que preconizem a formacao
do curso aproximando o aluno do mundo do trabalho com preceptoria e supervisao docente."""
""Orienta o desenvolvimento do estagio curricular ao longo da formacao, desconsiderando que se trata do mome
""desrespeita as orientacoes do proprio CNE/CES contidas no Parecer/CNE no 334/2019, o que representa um ret
r a substituicao de atividades presenciais relacionadas com a avaliacao, processo seletivo, Trabalho de Conclusao c
E/CP no 2/2021, considera a necessidade premente de retorno a presencialidade das atividades de aprendizado en

ais de estagios e de laboratorio dos demais cursos"""
s de graduacao presenciais"""

cionadas
a forma estipulada pela instituicao de ensino."""

sso de formacao e ainda sem o acumulo de experiencia necessaria para enfrentar essa pandemia"""
zada como substituta de horas devidas em sede de estagio curricular obrigatorio, nao os desobrigando de cumprir
gem e Fisioterapia, desde que o aluno, observadas as regras a serem editadas pelo respectivo sistema de ensino,"""
epcional, a anteciparem a colacao de grau dos alunos regularmente matriculados no ultimo periodo dos cursos de

carater excepcional, a anteciparem a colacao de grau dos alunos regularmente matriculados no ultimo periodo dc
os os Equipamentos de Protecao Individual"""
cial, deverao ser adotadas e normatizadas, para essa modalidade, atividades referentes ao TCC, avaliacao, extensao

ao da substituicao de que trata o caput deve obedecer as Diretrizes Nacionais Curriculares aprovadas pelo Conselho
er CNE no 005/2020"" (p. 6)"
eguranca dos colaboradores e para a higiene dos espacos. ""(p. 17)"
ra essa modalidade, atividades referentes as disciplinas praticas, inclusive de laboratorio, estagios, ao TCC, "" (p. 10
instituicao de educacao superior podera antecipar a conclusao dos cursos superiores"""
de do ensino presencial, pois e impossivel ensinar a cuidar sem o toque, sem acolher a dor e os medos na pratica JU

o Superior (IES) possuem autonomia para definir seus calendarios academicos, desde que respeitada a pertinente l
vidades referentes as disciplinas praticas, inclusive de laboratorio, estagios, ao TCC"" (p. 88)"
s, a aplicacao da excepcionalidade de que trata o caput deve obedecer as Diretrizes Nacionais Curriculares"""

resenciais relacionadas a avaliacao, processo seletivo, Trabalho de Conclusao de Curso (TCC)"" (p. 15)"
na formacao em detrimento a retomadas que possam apenas suprimir o potencial humano em falta nos servicos c
a do momento da expressao das competencias desenvolvidas ao longo do curso e que asseguram a terminalidade c
critica, reflexiva, fundamentada nas evidencias

nto da aprendizagem da construcao teorico-pratica da autonomia quanto as competencias desenvolvidas ao longo
rocesso tecnico, cientifico, politico e social em relacao as proprias DCN ainda vigentes (2001)."""
de Curso (TCC) e aulas de laboratorio, por atividades nao presenciais"""
n todos os niveis, etapas ou modalidades de ensino"""

carga horaria prevista para outras areas do estagio curricular obrigatorio, nos termos do Â§ 6o do art. 2o da Portar
"
Medicina, Enfermagem, Farmacia e Fisioterapia, desde que completada setenta e cinco por cento da carga horaria

os cursos de Medicina, Enfermagem, Farmacia e Fisioterapia, desde que completada setenta e cinco por cento da ca

o,

o Nacional de Educacao - CNE, ficando vedada a substituicao daqueles cursos que nao estejam disciplinados pelo CI

))"

JNTO ao usuario, a fim de que sejam desenvolvidos o senso critico,

legislacao, e

de saude"""
da formacao"""

do curso que se consolidam no final da formacao"""

ria GM/MEC no 356 de 20 de marco de 2020"""

prevista para o periodo de internato medico ou estagio supervisionado"""

arga horaria prevista para o periodo de internato medico ou estagio supervisionado,"""

NE"""
