# Supplementary material for: Consequences of the legislation issued for nursing education in times of COVID-19 (2020-2022)
Source: Rev Bras Enferm. 2024 Oct 7;77(5):e20230375. doi: 10.1590/0034-7167-2023-0375 (PMC11458146; doi:10.1590/0034-7167-2023-0375)
Supplement: Translation

# Translation: Consequências da legislação editada para o ensino de enfermagem em tempos de COVID-19 (2020-2022)

**DOI:** 10.1590/0034-7167-2023-0375pt

**Authors:** Ailla Gabrielli Costa Silva, Lenira Maria Wanderley Santos de Almeida, Regina Maria dos Santos, Danielly Santos dos Anjos Cardoso

**Affiliations:** I Universidade Federal de Alagoas. Maceió, Alagoas, Brasil

**Keywords:** Educação em Enfermagem, COVID-19, Política de Educação Superior, Legislação, Educação Superior

## Abstract

**Objetivos:**

analisar a legislação editada durante a pandemia de COVID-19 para o ensino em enfermagem e consequências imediatas.

**Métodos:**

pesquisa documental, cuja fonte foi a legislação editada para o ensino em enfermagem na pandemia de COVID-19. Foram analisados 32 documentos, submetidos a instrumento de coleta.

**Resultados:**

revelaram-se contradições e consequências para o ensino em enfermagem seguindo as diretrizes e lei de diretrizes e bases. As consequências a partir da lei incidiram sobre a qualidade do ensino, igualdade de acesso, dispensa de mínimo de dias letivos e abreviação de curso. Sobre as diretrizes, observou-se falta de condições de desenvolvimento de competências, além da tentativa de atualizá-la. Acerca dos estágios, a atuação na pandemia pôs os estudantes em risco, e sua abreviação impediu consolidarem conhecimentos e habilidades.

**Considerações Finais:**

o ensino remoto não garantiu a qualidade e igualdade de ensino, enfraqueceu o desenvolvimento de competências, não contemplando as necessidades educacionais da enfermagem.

## INTRODUÇÃO

Com a declaração da pandemia de *Coronavirus Disease* 2019 (COVID-19) pela Organização Mundial da Saúde em 2020, foi necessária a adoção de medidas, como o distanciamento físico, isolamento social e quarentena para prevenção e controle da transmissão do vírus. O distanciamento social requereu atividades não presenciais para os serviços considerados não essenciais, tais como aulas presenciais nas instituições de ensino, atividades práticas e estágios. Em resposta às medidas de distanciamento sociais adotadas e pela necessidade de continuidade das atividades, o Ministério da Educação (MEC) regulamentou o uso de tecnologias e ferramentas digitais para realização das aulas^(1)^.

Anteriormente à pandemia, Tecnologias Digitais de Informação e Comunicação (TDIC) já eram utilizadas, especialmente nos cursos de Educação a distância (EAD). Essa modalidade é regulamentada desde 2017 e se caracteriza pela mediação tecnológica entre professores e estudantes, com destaque para as atividades ocorrendo em lugares e tempos diversos^(1)^. Durante a pandemia, o que ocorreu foi a aplicação do ensino remoto emergencial, que se diferencia da EAD, por ser a transposição das aulas presenciais para o meio virtual, demandando, inclusive, regulamentação por parte do governo^(2)^.

O ensino superior é regido, no Brasil, pela Lei de Diretrizes e Bases da Educação Nacional (LDB), promulgada em 1996, e que traz as bases do ensino no Brasil, como o ensino superior. As Instituições de Ensino Superior (IES) elaboram e executam a proposta pedagógica com base nas diretrizes nacionais^(3)^. As Diretrizes Curriculares Nacionais do Curso de Graduação em Enfermagem (DCNEnf) foram publicadas em 2001 e trazem competências gerais e específicas a serem desenvolvidas durante a graduação pelo estudante. Preconizam a formação integral, pautada em metodologias ativas e de profissionais crítico reflexivos voltados ao Sistema Único de Saúde (SUS)^(4)^.

A aplicação do ensino remoto para a enfermagem exigiu que a substituição das aulas por meios virtuais fosse normatizada, tendo em vista que não era previsto na LDB ou nas DCNEnf o uso de TDIC de forma substitutiva ao ensino presencial^(1)^. Assim, a relevância deste estudo se traduz na possibilidade de produção de conhecimento acerca das circunstâncias da formação de trabalhadoras/es de saúde em meio à pandemia, com o registro sistematizado de possíveis consequências para o ensino de graduação em enfermagem.

Dessa forma, o estudo se guiou pela seguinte questão norteadora: que normativas foram instituídas para o ensino de graduação em enfermagem decorrentes das medidas governamentais e institucionais adotadas durante a pandemia de COVID-19 e suas consequências imediatas para a preservação da qualidade da formação profissional, tomando como base a LDB e as DCNEnf?

## OBJETIVOS

Analisar a legislação editada durante a pandemia de COVID-19 para o ensino em enfermagem e consequências imediatas.

## MÉTODOS

### Aspectos éticos

Como se trata de estudo documental cujas fontes são de domínio público e de livre acesso, não houve apreciação por parte de Comitê de Ética em Pesquisa, embora os princípios éticos tenham sido respeitados em toda a trajetória do trabalho.

### Tipo de estudo

Trata-se de pesquisa descritiva e documental com abordagem qualitativa, sendo um tipo de estudo em que as informações provêm de documentos, com o objetivo de compreender um fenômeno, e que se realiza em duas fases: análise preliminar do contexto e análise em si de seu conteúdo^(5)^. O *corpus* documental da pesquisa constituiu-se de 32 documentos normativos sobre o ensino durante a pandemia. Seguiram-se as recomendações do *checklist Standard for Reporting Qualitative Research* (SRQR), instrumento da *Enhancing the QUAlity and Transparency Of health Research* (Equator).

### Procedimentos metodológicos

As fontes de informações foram identificadas nos sites oficiais de órgãos e instituições, como Governo Federal, Ministério da Saúde, MEC, Conselho Nacional de Saúde (CNS), Conselho Nacional de Educação (CNE), Associação Brasileira de Enfermagem (ABEn) e Conselho Federal de Enfermagem (COFEN). Os órgãos e instituições identificados foram incluídos por emitirem normas legais, pareceres e recomendações para o ensino em geral e em enfermagem. A busca foi realizada no período de outubro de 2022 a abril de 2023. Os documentos foram buscados pelas palavras-chave “Educação a Distância/Remota/Emergencial”, “Ensino/Educação Superior”, “Ensino de/em Enfermagem”.

Inicialmente, foram encontradas 68 normativas, entre leis, resoluções, portarias, recomendações e pareceres. Em seguida, os documentos encontrados foram submetidos aos critérios de inclusão, como atendimento à pergunta de pesquisa e aos objetivos, além do recorte temporal da pandemia. Para serem incluídos, os documentos precisavam estar datados, assinados por autoridade e estabelecer normas ou recomendações a serem obedecidas ou observadas. Além disso, foram excluídas normativas de prorrogação, por não acrescentarem novos elementos ao ensino na pandemia, e leis que não tratassem do ensino superior e/ou ensino em enfermagem. O recorte temporal tomou como base a declaração da COVID-19 como uma emergência em saúde pública de importância internacional e o encerramento da emergência entre 03 de fevereiro de 2020 e 22 de abril de 2022^(6)^.

Os documentos selecionados foram submetidos a um instrumento de coleta de dados construído com base nos pontos abordados na LDB e nas DCNEnf. O instrumento coletou informações sobre tipo de documento, órgão, data de publicação, descrição do documento, palavras-chave, correlação e obediência à LDB (artigos da lei e trechos dos documentos), correlação e respeito às DCNEnf (tópicos da diretriz e trechos dos documentos). As informações extraídas por meio do instrumento foram organizadas em planilha do programa *Microsoft Excel®.*

Os documentos foram catalogados e sistematizados, conforme o conteúdo propusesse modificações à LDB ou às DCNEnf que repercutissem na qualidade da formação profissional com consequências imediatas à formação no recorte temporal. O resultado desta análise foi confrontado com a literatura, fazendo um diálogo com as normativas e gerando três categorias de análise.

## RESULTADOS

A partir da análise dos 32 documentos públicos selecionados, foram organizadas as seguintes categorias: Normativas relativas à Lei de Diretrizes e Bases da Educação Nacional e suas consequências (Subcategorias Aspectos gerais do ensino e Educação superior); Normativas relativas às Diretrizes Curriculares Nacionais do Curso de Graduação em Enfermagem e suas consequências; e Normativas para o estágio supervisionado e suas consequências. Após análise dos documentos, conseguiu-se inferir relações existentes entre eles, apresentadas em forma de mapa conceitual na Figura 1.

**Figura 1:** Relações existentes entre as normativas

### Normativas relativas à Lei de Diretrizes e Bases da Educação Nacional e suas consequências

#### Subcategoria 1: Aspectos gerais do ensino

A análise das normativas editadas para o ensino em enfermagem se inicia com a discussão sobre a LDB de 1996, já em seu Artigo (Art.) 1º, em que muitas das normativas trouxeram somente a vinculação da educação com o mundo do trabalho. A Portaria nº 356/2020/MEC, ao priorizar a inserção de estudantes no combate à pandemia, pontuou somente a necessidade do mercado, ignorando que os estudantes estavam em formação e que, além de estarem em risco, não forneceriam atendimento seguro e adequado à população, como reforçado pela ABEn, ao se posicionar contrária ao MEC^(3, 7, 8)^.

Art. 1º Fica autorizada aos alunos regularmente matriculados [...] do último ano dos cursos de enfermagem [...] a possibilidade de realizar o estágio curricular obrigatório em unidades básicas de saúde, unidades de pronto atendimento, rede hospitalar e comunidades a serem especificadas pelo Ministério da Saúde, enquanto durar a situação de emergência de saúde pública decorrente do COVID-19 (coronavírus)^(7)^.

Os princípios do ensino nacional, especialmente a garantia do padrão de qualidade, foram bastante recorrentes, seja por defender a sua manutenção ou ainda pela possibilidade de afetar a qualidade do ensino preconizada pela LDB. Essa preocupação foi mencionada especialmente nos pareceres publicados pelo CNE como defesa do uso de metodologias digitais. A substituição de aulas presenciais por aulas em meio digital não trouxe condições que assegurassem o cumprimento dessas finalidades com a substituição de aulas, como foi recomendado^(3, 9, 10, 11)^:

Importante salientar a manifestação do CNE em sua Nota de que, no processo de reorganização dos calendários escolares, deve ser assegurado que a reposição de aulas e a realização de atividades escolares possam ser efetivadas de forma que se preserve o padrão de qualidade previsto no inciso IX do artigo 3º da LDB e no inciso VII do artigo 206 da Constituição Federal^(10)^.

Outro ponto abordado dos princípios foi acerca da igualdade de condições de acesso e permanência na escola. Com a aplicação em larga escala do ensino remoto, iniciou-se o debate sobre as condições de acesso dos discentes a equipamentos e internet de qualidade. Isso é evidenciado na recomendação nº 48 do CNS, ao trazer que a aplicação, mesmo que teórica, da modalidade EAD não incluiu estudantes em vulnerabilidade^(3, 12)^.

Mesmo no caso das atividades teóricas, é necessário garantir as diferentes necessidades dos alunos. A imprecisão naquela norma legal sobre a que recursos, especificamente, se refere o escopo da mesma e sobre as formas de acompanhamento das atividades na modalidade EaD, que necessitam de equipamentos eletrônicos, inviabiliza a inclusão de estudantes que residem em áreas de maior vulnerabilidade e que não tenham recursos financeiros^(12)^.

O Art. 9º foi observado na regulamentação do governo federal, pois a União deve organizar, manter e desenvolver as IES, além de coletar, analisar e divulgar informações sobre educação, o que foi abordado no Ofício Circular nº 2/2020/CGLNES/GAB/SESU/SESU-MEC. Por fim, foi cumprido o papel da união ao baixar normas gerais sobre cursos de graduação^(3, 13)^. O Art. 9° também menciona o CNE como órgão de função normativa e de supervisão e atividade permanente. Ponto que foi cumprido e pôde ser observado nos pareceres emitidos e na Lei nº 14.040, que delegou ao CNE as diretrizes de implementação, posteriormente efetivou-se com o Parecer CNE/CP nº 1 1/2020^(3, 10, 11, 14)^.

O Art. 12º foi abordado pelas normativas federais na garantia de que as IES pudessem elaborar e executar sua proposta pedagógica. A recuperação dos estudantes pôde ser vista no Parecer nº 5/2020 do CNE, constando que as IES deveriam considerar “construir um programa de recuperação, caso necessário, para que todas as crianças possam desenvolver”^(3, 10)^. Os docentes também cumpriram o Art. 13° no que estavam incumbidos, sendo os Pareceres CNE/CP n° 5/2020 e n° 11/2020 alguns dos documentos que contiveram os pontos do referido artigo e o papel docente^(3, 10, 11)^.

#### Subcategoria 2: Educação superior

Adentrando o capítulo específico para o ensino superior, no Art. 43°, estavam descritas as finalidades da educação superior. Uma delas, a formação de diplomados, pôde ser entendida como justificativa para a Medida Provisória (MP) nº 934/2020, que permitiu abreviar cursos. O estímulo ao conhecimento de problemas do mundo presente foi observado com a promoção da campanha de prevenção no Ofício Circular nº 2/2020/CGLNES/GAB/SESU/SESU-MEC^(3, 13, 15)^.

As atividades não presenciais para reorganização do calendário encontraram base no Art. 47°. A Portaria nº 343/2020/MEC utilizou a necessidade de cumprimento dos dias letivos como justificativa para permitir a substituição de aulas em meios digitais: “As atividades acadêmicas suspensas deverão ser integralmente repostas para fins de cumprimento dos dias letivos e horas-aulas estabelecidos na legislação em vigor”^(3, 9)^.

No entanto, a MP nº 934/2020 posteriormente infringiu a prescrição do MEC, ao desobrigar o cumprimento de 200 dias mínimos letivos, sendo necessário somente cumprir a carga horária (CH) mínima, conforme instituído pela Lei nº 14.040/2020. A MP nº 934/2020 também alterou os requisitos para abreviação de cursos, estabelecendo que bastaria cumprir 75% da CH destinada ao estágio, como se pode ver^(3, 14, 15)^:

As instituições de educação superior ficam dispensadas, em caráter excepcional, da obrigatoriedade de observância ao mínimo de dias de efetivo trabalho acadêmico [...]. Na hipótese de que trata o caput, a instituição de educação superior poderá abreviar a duração dos cursos de [...], desde que o aluno, observadas as regras a serem editadas pelo respectivo sistema de ensino, cumpra, no mínimo [...] II - setenta e cinco por cento da carga horária do estágio curricular obrigatório dos cursos de enfermagem^(15)^.

A autonomia das universidades do Art. 53º foi garantida na maioria das atribuições, com exceção da adesão à ação “O Brasil conta comigo”, que ocorreu de maneira obrigatória, devendo as IES comunicar aos estudantes, sendo sugestão do CNS a participação facultativa entre IES e entes federativos. As atribuições foram feitas sob gestão democrática, conforme ordena o Art. 56º^(3, 16, 17)^.

Conforme Art. 55º, compete à União assegurar recursos suficientes para manutenção e desenvolvimento das IES que mantém, sendo definidas no Art. 70º as despesas para manutenção e desenvolvimento do ensino. Sobre fornecer equipamentos necessários ao ensino, o CNS, em sua recomendação nº 24/2020, propôs que as IES garantissem Equipamentos de Proteção Individual (EPIs) aos estudantes participantes da ação “O Brasil conta comigo”^(3, 17)^.

A postura do Governo Federal de imediatamente optar pelo ensino remoto logo no início da pandemia tem sua relação com o Art. 80º da LDB, que afirma que o poder público “incentivará o desenvolvimento e a veiculação de programas de ensino a distância, em todos os níveis e modalidades de ensino, e de educação continuada”. Esse fato explica a primeira decisão do MEC ser a substituição de aulas presenciais por aulas em meios digitais na Portaria nº 343/2020/MEC^(3, 9)^.

### Normativas relativas às Diretrizes Curriculares Nacionais do Curso de Graduação em Enfermagem e suas consequências

As DCNEnf são quem orientam a formação em enfermagem no país, trazendo competências a serem desenvolvidas, o que ocorre à medida que os estudantes têm contato com os cenários de prática. E o que se viu nas normativas foi a aplicação em larga escala do ensino remoto, em contradição ao que as entidades defendiam sobre a formação em enfermagem. Então, os documentos do MEC e CNE que instituíram aplicação de metodologias não presenciais para aulas, práticas e estágio curricular acabaram por interferir no desenvolvimento de competências necessárias aos estudantes, conforme afirmado pela ABEn e pelo CNS^(4, 9, 10, 18)^.

Ao Ministério da Educação (MEC), cobramos a observância das Diretrizes Curriculares Nacionais (DCNs) para formação de profissionais de enfermagem com a modalidade do ensino presencial, pois é impossível ensinar a cuidar sem o toque, sem acolher a dor e os medos na prática JUNTO ao usuário, a fim de que sejam desenvolvidos o senso crítico, a observação minuciosa, a realização dos procedimentos e a atitude ética e resolutiva^(18)^.

Entretanto, mesmo em condições de pandemia ou outra emergência sanitária, os recursos virtuais não suprem completamente o trabalho em saúde e, portanto, não abarcam as condições em que a maior parte das habilidades e competências profissionais devem ser desenvolvidas nas atividades de estágio, em situações reais^(12)^.

O próximo ponto são os conteúdos curriculares, que explicitam os conteúdos que devem ser abordados durante a graduação para promover o desenvolvimento das competências. A Lei nº 14.040, ao dispensar o mínimo de dias letivos, condicionou ao não prejuízo dos conteúdos essenciais à profissão, atuando de forma a promover o ensino preconizado quando se a analisa somente^(4, 14)^:

As instituições de educação superior ficam dispensadas, em caráter excepcional, da obrigatoriedade de observância do mínimo de dias de efetivo trabalho acadêmico [...], desde que [...] II - não haja prejuízo aos conteúdos essenciais para o exercício da profissão^(14)^.

Em 2021, o CNE apresentou à ABEn e ao CNS nova proposta de DCNEnf. De acordo com ofícios publicados pela ABEn e COFEN, além de manifesto da ABEn e moção do CNS, a proposta descaracterizou a graduação de enfermagem. Os documentos apontaram erros conceituais, esvaziaram conceitos, possibilidade de modalidade híbrida, não definição de estágio curricular e atividades práticas, além da falta de claro referencial teórico. Observou-se nos documentos a defesa pelo ensino de enfermagem de qualidade que permitiria o desenvolvimento das competências, com referência ao SUS, papel educativo e social do enfermeiro^(4, 19, 20, 21, 22)^.

A pesquisa e a extensão fazem parte do rol de atividades complementares reconhecidas pelas DCNEnf, sendo recomendada a realização virtual das pesquisas e a regulamentação das atividades de extensão, com sugestão de associação da pesquisa e extensão à substituição do estágio^(11)^.

A organização do curso é assegurada nas DCNEnf com a construção de projeto pedagógico, com formação integral, articulação entre ensino, pesquisa, extensão e assistência, e metodologias que articulem o aprender a aprender e aprender a fazer. O próprio CNE, no Parecer nº 19/2020, reconheceu que a substituição por aulas digitais representou uma limitação no uso de metodologias e tecnologias voltadas ao virtual. A possibilidade de supressão de práticas profissionais acabou por interferir na interação dos tutores e estudantes com a população, já que a prática favorece a integração teoria-prática durante o curso. Acerca do tópico de acompanhamento e avaliação, o Parecer CNE/CP nº 5/2020 trouxe a necessidade de avaliação do ensino no período^(4, 10, 23)^.

Por fim, o Trabalho de Conclusão de Curso (TCC) é normatizado nas DCNEnf, em organização do curso, como requisito para conclusão do curso, elaborando um trabalho sob orientação docente. O CNE orientou que as atividades relacionadas ao TCC ocorressem de forma não presencial, sendo necessária sua regulamentação^(4, 10)^.

### Normativas para o estágio supervisionado e suas consequências

A última categoria identificada corresponde ao estágio supervisionado, que perpassa as duas normativas. Os estágios terão suas normas de realização estabelecidas pelos sistemas de ensino segundo o Art. 82º da LDB, obedecendo à lei federal. Nas DCNEnf, há a obrigação da inclusão do estágio em serviços de saúde com no mínimo 500 horas nos últimos dois semestres. Inicialmente, o estágio foi proibido de ser substituído por meios digitais, com a Portaria nº 343/2020/MEC, passando para a realização como atuação no combate à pandemia, com a Portaria nº 356/2020/MEC^(3, 4, 7, 9)^.

A ABEn prontamente se manifestou contrária ao uso dos estudantes como força de trabalho, assim como o CNS. O governo federal, através da MP nº 934/2020, permitiu a abreviação do curso com a conclusão de 75% da CH do estágio obrigatório. O CNE, por meio do Parecer CNE/CP nº 5/2020, se posicionou favorável à adoção de atividades não presenciais para os estágios, o que foi acatado pelo MEC na Portaria nº 544/2020/MEC^(8, 10, 15, 17, 24)^.

## DISCUSSÃO

O primeiro ponto a se analisar é a diversidade de nomenclaturas utilizadas, como EAD, aulas em meio digitais, atividades remotas, TDIC, aprendizagem e ensino remoto. Os termos “EAD” e “ensino remoto”, que, por vezes são usados como sinônimos, referem-se a modalidades distintas. EAD tem como característica a sistematização do ensino-aprendizagem, metodologia, estratégias e materiais específicos planejados; modelo voltado para estudantes, professores e tutores; acompanhamento pelos professores ou tutores; atividades síncronas e assíncronas; uso de recursos em plataformas diversas. O ensino remoto emergencial se caracteriza pelo distanciamento físico de estudantes e professores, caráter temporário e principalmente pela transição do ensino presencial para o virtual com o objetivo de mitigar impactos e prejuízos na aprendizagem dos alunos^(2, 25)^.

A LDB e as DCNEnf regulamentam e trazem os princípios para o padrão de qualidade do ensino nacionalmente. Fernandes *et al.* ^(26)^, ao analisarem os atos federais normativos adotados durante a pandemia, refletiram que por vezes houve a limitação ou não foi contemplada a execução de tarefas essenciais que resultassem em qualidade. Apontaram contrariedades nas portarias publicadas, que dispensaram o mínimo de dias letivos desde que cumprida a CH e, ao mesmo tempo, permitiram abreviação de cursos. Outra contradição foi a aplicação do ensino remoto para proteger estudantes e profissionais enquanto os convocavam para atuar na pandemia. Os autores argumentaram que o ensino remoto não trouxe condições para cumprimento das finalidades do ensino instituídas pela LDB, e ainda contrariam as DCNEnf quando permitiram atividades práticas a distância.

O estudo de Fernandes *et al.* ^(26)^ avaliou que as medidas visaram suprir profissionais para os serviços de saúde sem se preocupar com a qualidade da formação. O ensino e a prática, aliados ao mundo de trabalho, oportunizam ao estudante a formação do pensamento crítico sobre seu desempenho e desenvolver habilidades. Tirar a formação da realidade, experienciada nas práticas, leva a uma formação somente teórica, sem problematizar questões sociais, não adotando o SUS como ordenador. Ao deixar a critério das IES disponibilizar ferramentas, não se considerou o orçamento das IES públicas, e não houve a responsabilização da União na garantia de recursos. Os autores concluíram que o ensino remoto não contemplou as necessidades de formação em enfermagem, repercutindo na manutenção da qualidade proposta pela LDB, além de abordar outros pontos da lei.

A igualdade de condições de acesso previstas na LDB avistou na excepcionalidade da pandemia uma situação nunca imaginada. Suspensão de aulas por longos períodos e mudança abrupta para o ambiente virtual esbarraram na desigualdade socioeconómica no país, já que especialmente os estudantes da rede pública têm dificuldade de acesso à internet e às tecnologias necessárias. Os impactos potencializaram as desigualdades já existentes, além de ocorrer a desnivelação de aprendizados. A urgência justificada nas medidas tomadas não deveria ser utilizada para transformar o direito de todos à educação no direito de poucos que possuem recursos^(27)^.

O acesso ao ensino não se dava somente em ter acesso à internet; também era necessário possuir equipamentos e alfabetização digital para uso das ferramentas adotadas. A democratização do acesso deveria estar vinculada a uma política de ampliação do acesso digital à população. Isso permitiria o uso adequado das tecnologias utilizadas, fato que não ocorreu e tampouco é discutido atualmente. Essas mudanças citadas afetam a todos, e, para o docente, há ainda a exigência e a sobrecarga de CH, gerando estresse ocupacional e afetando a saúde física e mental^(28)^.

O CNE, como órgão de função normativa, de supervisão e atividade permanente nas atividades educacionais, ganhou destaque com os pareceres regulamentando o ensino na pandemia. Mascarenhas e Franco^(29)^ analisaram o Parecer nº 5/2020, que foi o primeiro a sugerir atividades não presenciais. O parecer se referiu à educação como o cumprimento de horas e conteúdos, o que se aplicava à Portaria nº 343/2020/MEC, desconsiderando o papel social da escola e expondo um caráter tecnicista adotado pelo governo, ignorando a situação real do ensino no país. As autoras consideraram, por fim, o parecer frágil, discriminatório e perverso com estudantes da rede pública.

A extensão, no contexto da pandemia, sofreu com as medidas de distanciamento social. Algumas foram inicialmente suspensas por serem inviáveis devido ao isolamento social. Durante a implantação do ensino remoto emergencial, houve aceleração na produção do ensino e pesquisa com adaptação para ações de combate à pandemia. As ações de combate envolveram testagem para COVID-19, materiais educativos, participação na campanha de vacinação, entre outros^(28, 30)^.

Um dos pontos que mais demandou regulamentação foi o estágio obrigatório. Os estágios curriculares na saúde demandam uma presencialidade que não era possível devido às condições impostas pela pandemia. As medidas adotadas permitiram o estágio de estudantes em formação na pandemia sem garantia de segurança ou do processo formativo. Posteriormente, foi permitida a substituição em meios digitais que constasse nas DCNEnf, aprovada pelo colegiado. Os atos legais demonstraram preocupação em suprir deficiências dos serviços, sem considerar outras dificuldades estruturais existentes, como falta de EPIs. Questões como segurança do paciente, assistência com menor ocorrência de erros e alinhamento com as IES também foram deixadas de lado^(31)^.

Os cenários de prática e de estágio precisam ser preservados como espaços de ensino essenciais na aprendizagem e na articulação teoria/prática, vista nas DCNEnf. A diversidade de cenários também contribui para a construção da identidade profissional, produção do cuidado e articulação de conhecimento com a realidade de saúde da população. A abreviação de cursos, mediante cumprimento de 75% da CH do estágio, também veio para acelerar a formação para suprir demandas do serviço. Ao mesmo tempo, impediu a consolidação dos conhecimentos teóricos, desenvolvimento de habilidades técnicas e atitudes para o futuro profissional do estudante^(31)^.

Analisando a ação “O Brasil conta comigo” e a abreviação do curso, Mata *et* al.^(32)^ resgataram que as diretrizes da formação orientam que os profissionais devem atender às necessidades sociais em saúde de forma integral e humanizada. A formação que alcançasse esse sucesso nos egressos já era considerada difícil antes da pandemia, devido à inconsistência do discurso com a prática, fato observado pelos alunos, inconsistência que tendia a se aprofundar com a antecipação da conclusão^(32)^. O ensino deve ser fortalecido, não enfraquecido, alinhando-se aos pressupostos teóricos do SUS, efetivando, assim, a formação generalista no lugar do foco no assistencialismo e nas hiperespecializações.

Mesmo antes da pandemia, a necessidade do estágio supervisionado já era reforçada como desenvolvedora das competências gerais. Benito *et al.* ^(33)^ descreveram as competências gerais das DCNEnf e as articularam com o estágio supervisionado. O desenvolvimento completo não se conclui durante a formação, sendo um compromisso do profissional, porém os autores apontaram o estágio e as práticas como fortalecimento, promoção e ampliação das competências, sendo a aprendizagem por competências um processo pedagógico de maior transformação que recebe as informações passivamente. Há também a atuação do estudante como ser provocador de mudanças que irá atuar moldando a realidade a ser enfrentada pela equipe de saúde.

Outros pontos a serem analisados não tiveram repercussão direta a partir das normativas, mas são questões inerentes à EAD e atualmente também ao ensino remoto para a enfermagem. Portanto, também cabe sua discussão, já que, mesmo não sendo a modalidade utilizada na pandemia, o uso de TDIC na enfermagem não é novo e vem sendo discutido há tempos por teóricos na área.

O ano de 2020 foi considerado o ano internacional da enfermagem, com o lançamento de campanhas como *Nursing Now,* que tiveram outra dimensão com a pandemia. Um dos pontos da campanha foi a valorização da profissão, em que se entende o enfermeiro como líder, com papel de excelência, realizando cuidado com ética e de forma qualificada. Assim, uma das formas de se valorizar e promover esse perfil é por meio do fortalecimento da educação e desenvolvimento profissional. No entanto, o que foi visto foram denúncias de péssimas condições de trabalho para a enfermagem, adoecimento de profissionais e a adoção massiva do ensino remoto na formação^(34)^.

A autonomia profissional está relacionada à incorporação da enfermagem enquanto ciência e do Processo de Enfermagem. Historicamente, a autonomia esteve ligada à formação, com Florence trazendo uma autoridade às enfermeiras dentro do seu campo do saber, até o momento em que a formação foi modificada para atender aos interesses financeiros e médicos. Quando conseguiu-se descaracterizar a enfermagem enquanto ciência, houve a perda de autonomia das enfermeiras, que se reduziram à reprodutora de ordens da classe médica. A enfermeira precisa se apoderar da sua ciência, estando diretamente relacionada a uma formação que promova a autonomia e a aplicação do Processo de Enfermagem. Além disso, há necessidade de legislação voltada à enfermagem e gestores que defendam a atuação profissional, para que se concretize a práxis da enfermeira, reconhecida e valorizada^(35)^.

Um problema gerado pela oferta de cursos em modalidade a distância está no avanço da privatização do ensino, levantando dúvidas sobre o comprometimento social, em especial a formação ordenada para o SUS, conforme orientado pela Constituição Federal de 1988, Lei Orgânica da Saúde e DCNEnf^(36)^.

A alternativa encontrada para o desenvolvimento de atividades práticas em laboratórios foi a substituição para aulas teóricas, não realização, ou a aula ocorrer com o docente no laboratório e o estudante em casa de forma síncrona. Os autores ainda trouxeram a diminuição do número de estudantes por grupo de prática, o que leva à incerteza na qualidade das atividades oferecidas, tanto em termos do impacto do incumprimento quanto da substituição da prática pela mediação tecnológica^(37)^.

Mesmo com a Lei Federal nº 14.040/2020 condicionando a isenção de jornadas letivas mínimas à manutenção da CH esperada e sem comprometimento de conteúdos, o que aconteceu na realidade foi diferente. Baixinho e Ferreira^(38)^ identificaram no discurso de estudantes que a redução de horas foi uma dificuldade encontrada durante o estágio, gerando apreensão em relação à capacidade de aprendizado e à tomada de decisão clínica, além do desenvolvimento profissional.

O cuidado em enfermagem exige a presença e a utilização dos sentidos, sendo conceito central em diversas teorias de enfermagem. Hoje, há o debate sobre a presença em um mundo digital que se estende também à enfermagem. O estar com o paciente, que engloba o toque, ser ouvinte e a compreensão, transformou-se durante a pandemia em um desafio à essência do cuidado. Se, para o enfermeiro, a presença é imprescindível para a realização do cuidado de enfermagem, por que seria diferente para o estudante? Assim como os profissionais, os estudantes, além de desenvolverem competências, necessitam de preparo para trabalhar com pessoas em qualquer circunstância da vida e ainda mais em condições de sofrimento, de modo que o ensino meramente cognitivo não alcança esse objetivo, nem uma prática segura, solidária, competente e ética. Isso leva à reflexão sobre a necessidade do ensino presencial e de exercer a presença como cuidado^(25, 39)^.

### Limitações do estudo

Ter sido realizado sob a ótica da pesquisa documental, restrito a normativas federais, a alguns órgãos e instituições, limitando a análise às questões legais, sem investigar a efetivação das mudanças, percepções do ensino ofertado e possíveis repercussões à saúde de docentes e estudantes.

### Contribuições para a área da enfermagem

As contribuições do estudo estão na disponibilização de relevante material sistematizado, além da análise sobre as repercussões da aplicabilidade dos instrumentos normativos para o ensino em enfermagem durante a pandemia, a partir da LDB e das DCNEnf.

## CONSIDERAÇÕES FINAIS

Pesquisadores do ensino em enfermagem apontam a larga aplicação do ensino remoto como fator promotor de fragilidades do desenvolvimento de competências e da consolidação de conhecimentos teóricos. Alguns dos aspectos relevantes que contribuíram para essa condição foram: a substituição dos cenários de prática e estágio obrigatório por tecnologias digitais; as distintas formas de acesso ao ensino remoto que estudantes e docentes foram submetidos, tanto pela necessidade de aquisição de equipamentos e internet de qualidade quanto pela estrutura, habilidades e conhecimentos adequados para o uso de plataformas e recursos; e a discussão sobre o padrão de qualidade da formação proposto pela LDB e sua real efetivação em meio à pandemia.

Além das medidas tomadas em decorrência da pandemia, houve a tentativa, por parte do CNE, da atualização das DCNEnf para a enfermagem, situação essa que mobilizou órgãos da enfermagem a apontar fragilidades teóricas, metodológicas, além da não possibilidade de implementação do ensino híbrido pela natureza da profissão e a ausência do SUS como ordenador da formação na proposta apresentada.

Cabe repensar o uso de TDIC como substitutas do ensino presencial, pela possibilidade de ocorrência de nova pandemia. Gestores e docentes devem trabalhar planos de incorporação das tecnologias como auxílio pedagógico, não em substituição, considerando que se trata de um aprender que requer contato, vínculo, toque e, portanto, desenvolve aprendizados de relações intersubjetivas de cuidados.

Por outro lado, é importante instituir comissões de biossegurança para construir uma ação coordenada a partir de protocolos que assegurem a proteção adequada ao ensino presencial e articular ações em que a universidade, através do curso de enfermagem, possa colaborar de modo seguro com a população, fazendo valer seu compromisso ético e social. Dessa forma, estará também cooperando com a familiaridade de docentes e discentes a ferramentas tecnológicas de ensino, sem limitar o desenvolvimento de competências e fortalecendo a construção de novas alternativas.

## Data Availability

https://doi.org/10.48331/scielodata.TSZQM9
